# Supplementary material for: Overexpression of RCAN1, a Gene on Human Chromosome 21, Alters Cell Redox and Mitochondrial Function in Enamel Cells
Source: Cells. 2022 Nov 11;11(22):3576. doi: 10.3390/cells11223576 (PMC9688881; doi:10.3390/cells11223576)
Supplement: Supplementary file 1 [file cells-11-03576-s001.zip › cells-1981147-supplementary.pdf]

Table S1. Primers sequences in RT-PCR

**Name**	**Sequence**
**Rat**	
Rcan1 F (rat)	ACGACACTCGGTGAACATT
Rcan1 R (rat)	GAACATCAACCCATTTGCTC
**Mouse**	
RCAN1.4 F	CTTGTGTGGCAAACGATGATG
RCAN1.4 R	TGGTGTCCTTGTCATATGTTCTG
RCAN1.1 F	TCGACTGCGTAGATGGAGG
RCAN1.1 R	TGGTGTCCTTGTCATATGTTCTG
Amelx (fw)	GTCACCTCTGCATCCCATG
Amelx (rev)	TTCCCGCTTGGTCTTGTC
Ambn (fw)	TGAGCCTTGAGACAATGAGAC
Ambn (rev)	AAAGAGTTATGCGGTGGGAG
Enam (fw)	TATGGTCTTCCACCAAGGAA
Enam (rev)	TAGGCACACCATCTCCAAAT
Mmp20 (fw)	CACCTCACAAGCCATCTATCC
Mmp20 (rev)	GAAGCTCCTTTCCCAACATTG
Klk4 (fw)	CAACTAAAGAATGGGAAACTGCC
Klk4 (rev)	AAGATTTGGGAGGCTAACGG
